# The endothelial nitric oxide synthase/cyclic guanosine monophosphate/protein kinase G pathway activates primordial follicles

**DOI:** 10.18632/aging.202235

**Published:** 2020-12-03

**Authors:** Peikun Zhao, Zidai Song, Yan Wang, Han Cai, Xiaoyan Du, Changlong Li, Jianyi Lv, Xin Liu, Meng Guo, Zhenwen Chen

**Affiliations:** 1 Beijing Key Laboratory of Cancer Invasion and Metastasis Research, School of Basic Medical Sciences, Capital Medical University, Beijing, People’s Republic of China; 2 Fujian Provincial Key Laboratory of Reproductive Health Research, Medical College of Xiamen University, Xiamen, Fujian, China

**Keywords:** eNOS/cGMP/PKG pathway, mTOR, FBXW7, primordial follicle activation, ubiquitination

## Abstract

In mammals, the well-organized activation of quiescent primordial follicles is pivotal for female reproductive reserve. In the present study, we examined the mechanisms underlying primordial follicle activation in mice. We found that endothelial nitric oxide synthase (eNOS) and its downstream effectors, cyclic guanosine monophosphate (cGMP) and cGMP-dependent protein kinase G (PKG), were expressed in pre-granulosa cells and promoted primordial follicle activation, oocyte growth and granulosa cell proliferation in neonatal ovaries. Mammalian target of rapamycin (mTOR) colocalized with PKG in pre-granulosa cells and was essential for eNOS/cGMP/PKG pathway-induced primordial follicle activation. The eNOS/cGMP/PKG pathway was found to stabilize mTOR protein. The mRNA levels of F-box and WD repeat domain containing 7 (FBXW7), an E3 ubiquitin ligase, correlated negatively with mTOR protein levels in neonatal ovaries. FBXW7 bound to and destabilized mTOR protein in pre-granulosa cells in a ubiquitin/proteasome-dependent manner. However, agonists of the eNOS/cGMP/PKG pathway reduced FBXW7 mRNA levels. FBXW7 overexpression suppressed primordial follicle activation and prevented the eNOS/cGMP/PKG pathway from activating primordial follicles and stabilizing mTOR protein. These findings demonstrate that the eNOS/cGMP/PKG pathway activates primordial follicles by suppressing FBXW7-induced ubiquitination of mTOR in mice.

## INTRODUCTION

For mammalian females, follicles are the basic structural and functional units of the ovaries, and provide fertilizable oocyte resources throughout the reproductive life-span [1–3]. Oocytes surrounded by several flattened pre-granulosa cells (preGCs) form primordial follicles (PFs), which are maintained in a quiescent state [4, 5]. However, beginning two to four days post-partum (dpp) in mice, a number of PFs are progressively and irreversibly recruited into the growing pool to generate mature oocytes [3, 6]. The equilibrium between the dormant pool and the growing pool ensures the proper fertile life-span in females [3]. Thus, abnormal activation of the PF reserve may severely impair fertility and cause primary ovarian insufficiency (POI) [7–9].

Appropriately regulated mammalian target of rapamycin (mTOR) signaling in preGCs is necessary for PF activation. In mice, knocking out the regulatory-associated protein of mTOR in preGCs impairs mTOR signaling and keeps PFs quiescent, while deleting the mTOR inhibitor tuberous sclerosis 1 in preGCs awakens nearly all PFs [10]. Rapamycin, a pharmacological inhibitor of mTOR, has been used to prevent the overactivation of PFs in multiple POI mouse models [11–13]. Previous studies have suggested that mTOR and KIT ligand (KITL) in preGCs activate KIT/phosphoinositide 3-kinase (PI3K)/AKT signaling and induce the nuclear export of forkhead box O3a (FOXO3a) in oocytes, which consequently awakens dormant oocytes and PFs [14–19]. While mTOR is known to be important for PF activation, the upstream signals of mTOR in preGCs remain unclear. Recently, AKT stimulators were applied for the *in vitro* activation of PFs in POI patients [20], and although healthy babies were delivered, the low success rate hinted that this approach needs to be improved. Thus, novel activators of PFs should be identified.

Endothelial nitric oxide (NO) synthase (eNOS) produces membrane-permeant NO and is involved in angiogenesis, blood pressure regulation and tumorigenesis [21, 22]. NO stimulates soluble guanylate cyclase (sGC), which generates the second messenger cyclic guanosine monophosphate (cGMP) and subsequently activates cGMP-dependent protein kinase G (PKG) [23]. eNOS is gonadotropin-sensitively expressed in oocytes and somatic cells of growing follicles (GFs), and is important for ovarian development [24, 25]. Knocking out eNOS was found to diminish fertility, oocyte meiotic maturation and ovulation in mice [25–27], while NOS substrate injection was reported to enhance follicle progression and AKT/FOXO3a activation in rats [28]. In addition, cGMP maintains oocyte meiosis arrest [29], and functions with PKG to reduce hormone release, increase glucose uptake and suppress apoptosis in granulosa cells [30–32]. Although NO and the downstream cGMP/PKG pathway are critical for ovarian development, it is unclear whether and how this pathway promotes PF activation.

Ubiquitin/proteasome-dependent protein degradation induced by E3 ubiquitin ligase is important for the maintenance of cellular protein homeostasis [33–36]. The E3 ubiquitin ligase F-box and WD repeat domain containing 7 (FBXW7), is a p53-dependent tumor suppressor [35], that degrades multiple oncoproteins. Notably, FBXW7 ubiquitinates and degrades mTOR, and loss or mutation of FBXW7 increases the sensitivity of cancer cells and mouse models to rapamycin [36–39]. FBXW7 only binds to the mTOR complex mTORC1 [40], which is coincidently the mTOR complex involved in PF activation [10]. However, it is not known whether FBXW7 ubiquitinates mTOR during follicle development.

In this study, we used an *in vitro* neonatal ovary culture system to investigate whether the NO-stimulated cGMP/PKG pathway was involved in PF activation, and whether these effects depended on the enhancement of mTOR expression. We also investigated whether FBXW7 influenced PF activation or the eNOS/cGMP/PKG pathway activity by ubiquitinating mTOR in mice.

## RESULTS

### eNOS promoted PF activation and oocyte growth

**Figure 1:**
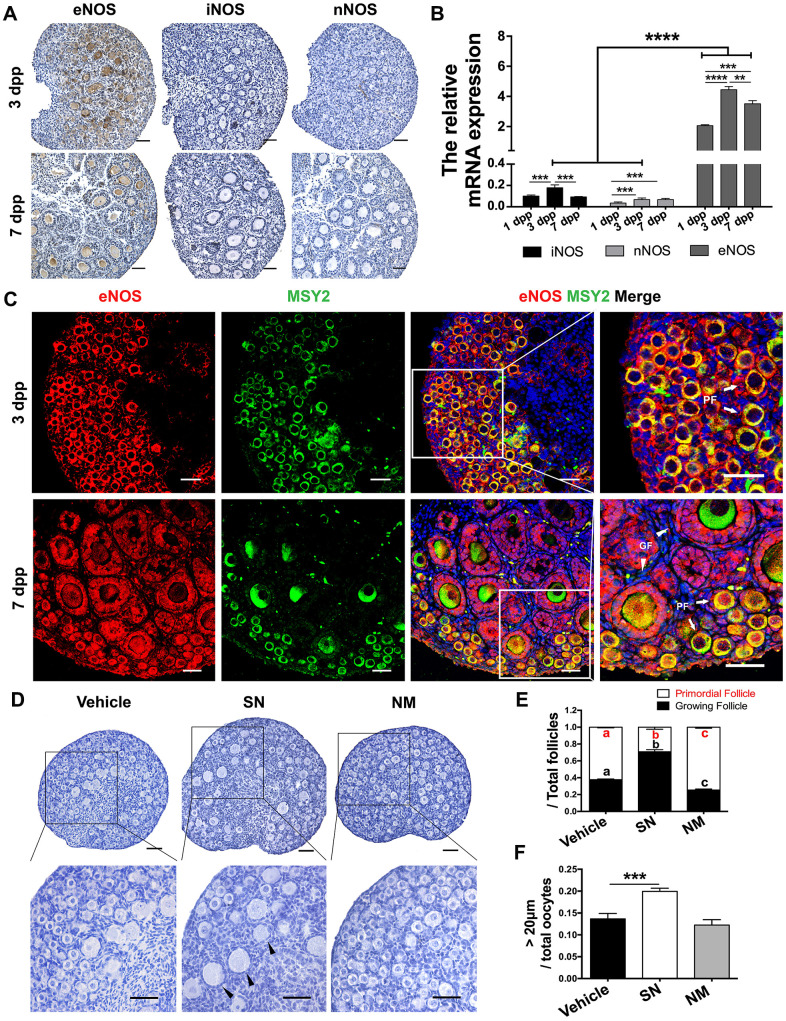
**eNOS was involved in PF activation.** (**A**) Immunohistochemical staining of eNOS, iNOS and nNOS in ovaries at 3 dpp and 7 dpp. Scale bar, 40 μm. (**B**) The relative mRNA levels of eNOS, iNOS and nNOS were assessed in ovaries from 1-7 dpp using qRT-PCR. (**C**) Immunofluorescence staining of eNOS in ovaries at 3 dpp and 7 dpp. Sections were labeled for eNOS (red), the oocyte marker Y box protein 2 (MSY2, green) and the nuclear marker Hoechst (blue). Arrows indicate PFs and triangles indicate GFs. Scale bar, 40 μm. (**D**) Ovaries at 1 dpp were treated with the vehicle, the NO donor SN (100 μM) or the NO inhibitor NM (1 mM) for six days (n=6). The ovarian morphology was analyzed after hematoxylin staining. Triangles indicate GFs. Scale bar, 40 μm. (**E**) The numbers of PFs and GFs/the total number of follicles were analyzed. (**F**) The numbers of oocytes with diameters larger than 20 μm were counted. **, *** and **** denote statistical significance at *p* < 0.01, *p* < 0.001 and *p* < 0.0001 respectively. Different letters with the same color denote statistical significance at *p* < 0.001 (Red letters represent the proportions of PFs, while black letters represent the proportions of GFs).

eNOS is important for ovarian development. Using immunohistochemistry, we found that eNOS was the only detectable NOS isoform in neonatal mouse ovaries (Figure 1A). To determine the effects of eNOS on PF activation, we examined its expression pattern and location in the neonatal ovaries. The mRNA levels of eNOS were 20 times higher than those of inducible NOS (iNOS) and neuronal NOS (nNOS). Moreover, eNOS levels increased significantly from 1 to 3 dpp, and remained relatively high thereafter (Figure 1B). Immunofluorescence staining indicated that eNOS was strongly stained in the cytoplasm of both oocytes and preGCs in PFs, and was predominantly expressed in the cytoplasm of granulosa cells in GFs (Figure 1C). These data implied that eNOS might be involved in neonatal ovarian development.

To further investigate the function of eNOS, we treated the ovaries at 1 dpp with the NO donor SNAP (SN) or the NOS inhibitor L-NMMA (NM) *in vitro* for six days. We found that the proportion of PFs decreased significantly after SN treatment, but increased significantly after NM treatment compared with vehicle treatment (Figure 1D, 1E). The NO donor also increased the proportion of oocytes with diameters larger than 20 μm (Figure 1F), suggesting that NOS promotes oocyte growth. The total numbers of oocytes and follicles did not differ significantly after NO signal was altered (Supplementary Figure 1). Inhibiting eNOS suppressed PF activation, while inhibiting the other two NOS isoforms (iNOS and nNOS) did not (Supplementary Figure 2). These results indicated that eNOS promotes PF activation and oocyte growth.

### The effects of eNOS on PF activation depended on cGMP/PKG

**Figure 2:**
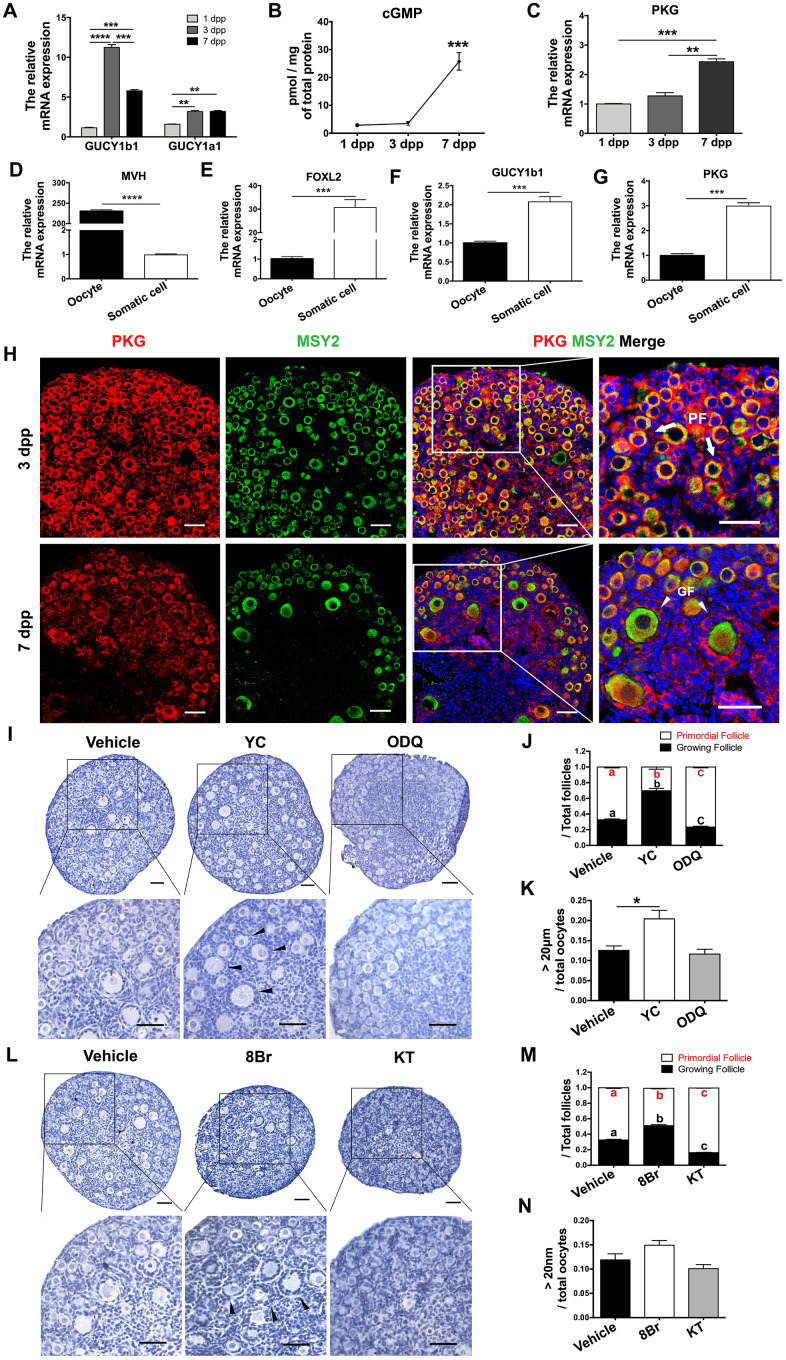
**cGMP/PKG participated in PF activation.** (**A**) The relative mRNA levels of GUCY1b1 and GUCY1a1 in ovaries from 1-7 dpp. (**B**) Radioimmunoassay of cGMP levels in ovaries from 1-7 dpp (n=20). (**C**) The relative mRNA levels of PKG in ovaries from 1-7 dpp. (**D**–**G**) The relative mRNA levels of (**D**) the germline marker DEAD box helicase 4 (MVH), (**E**) the granulosa cell marker forkhead box L2 (FOXL2), (**F**) GUCY1b1 and (**G**) PKG in oocytes and somatic cells isolated from ovaries at 3 dpp. (**H**) Immunofluorescence staining of PKG and MSY2 in ovaries at 3 dpp and 7 dpp. Arrows indicate PFs and triangles indicate GFs. Scale bar, 40 μm. (**I**, **L**) Ovaries at 1 dpp were treated with the vehicle, the sGC agonist YC (10 μM), the sGC inhibitor ODQ (1 μM), the cGMP analog 8Br (10 μM) or the PKG inhibitor KT (1 μM) for six days (n=6). The ovarian morphology was analyzed after hematoxylin staining. Triangles indicate GFs. Scale bar, 40 μm. (**J**, **M**) The numbers of PFs and GFs/the total number of follicles were analyzed. (**K**, **N**) The numbers of oocytes with diameters larger than 20 μm were counted. *, ** and *** denote statistical significance at *p* < 0.05, *p* < 0.01 and *p* < 0.001, respectively. Different letters with the same color denote statistical significance at *p* < 0.01 (Red letters represent the proportions of PFs, while black letters represent the proportions of GFs).

cGMP and PKG are known to be major downstream signals of NOS [41, 42]. Thus, we explored the involvement of cGMP/PKG in PF activation. The mRNA levels of the two major sGC subunits (GUCY1a1 and GUCY1b1) peaked in the ovaries at 3 dpp (Figure 2A), following the tendency of eNOS mRNA levels. After the increases in eNOS and sGC, the levels of cGMP and PKG mRNA both increased significantly at 7 dpp (Figure 2B, 2C). We also isolated oocytes and somatic cells from the ovaries at 3 dpp, as reported previously [43]. Quantitative real-time PCR (qRT-PCR) indicated that GUCY1b1 and PKG were predominantly expressed in somatic cells (Figure 2D–2G). Consistently, immunofluorescence staining demonstrated that PKG was mainly expressed in the cytoplasm in both preGCs from PFs and granulosa cells from GFs (Figure 2H).

**Figure 3:**
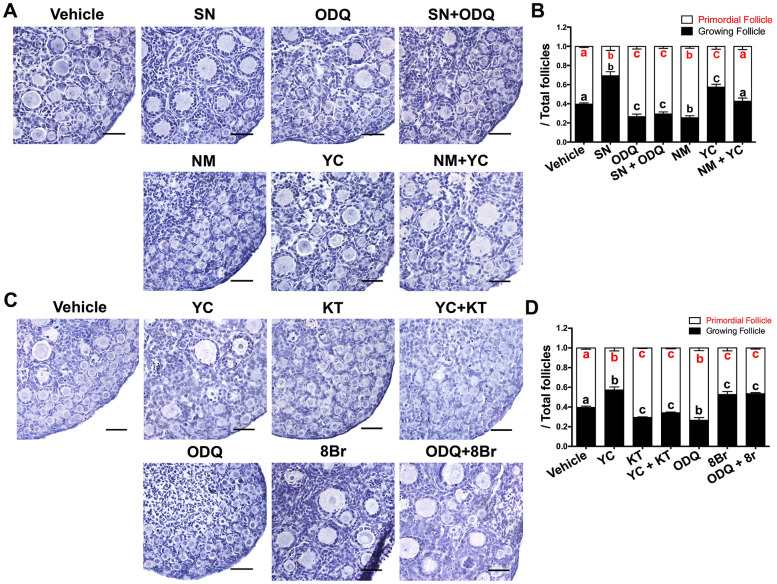
**The activation of PFs by eNOS depended on cGMP/PKG.** (**A**, **B**) Ovaries at 1 dpp were treated with the vehicle, SN (100 μM), ODQ (1 μM), SN + ODQ, NM (1 mM), YC (10 μM) or NM + YC for six days (n=6). (**A**) The ovarian morphology was analyzed after hematoxylin staining. (**B**) The numbers of PFs and GFs/the total number of follicles were analyzed. (**C**, **D**) Ovaries at 1 dpp were treated with the vehicle, YC (10 μM), KT (1 μM), YC + KT, ODQ (1 μM), 8Br (10 μM) or ODQ + 8Br for six days (n=6). (**C**) The ovarian morphology and (**D**) the numbers of PFs and GFs/the total number of follicles were analyzed. Scale bar, 40 μm. Different letters with the same color denote statistical significance at *p* < 0.01 (Red letters represent the proportions of PFs, while black letters represent the proportions of GFs).

Next, we used various agonists and inhibitors to further examine the effects of cGMP/PKG on PF activation. The proportions of GFs and oocytes with diameters larger than 20 μm increased in ovaries treated with the sGC agonist YC-1 (YC) or the cGMP analog 8-Br-cGMP (8Br), but decreased significantly in ovaries treated with the sGC inhibitor ODQ or the PKG inhibitor KT-5823 (KT) (Figure 2I–2N), illustrating that cGMP and PKG activate PFs. In addition, ODQ prevented the NO donor SN from activating PFs (Figure 3A, 3B), and KT blocked the actions of YC (Figure 3C, 3D). The suppression of PF activation by the NOS inhibitor NM was reversed by YC (Figure 3A, 3B), and the suppression of PF activation by ODQ was reversed by 8Br (Figure 3C, 3D). These data indicated that the effects of eNOS on PF activation depend on the cGMP/PKG pathway in preGCs.

### The eNOS/cGMP/PKG pathway promoted oocyte FOXO3a translocation and granulosa cell proliferation during PF activation

**Figure 4:**
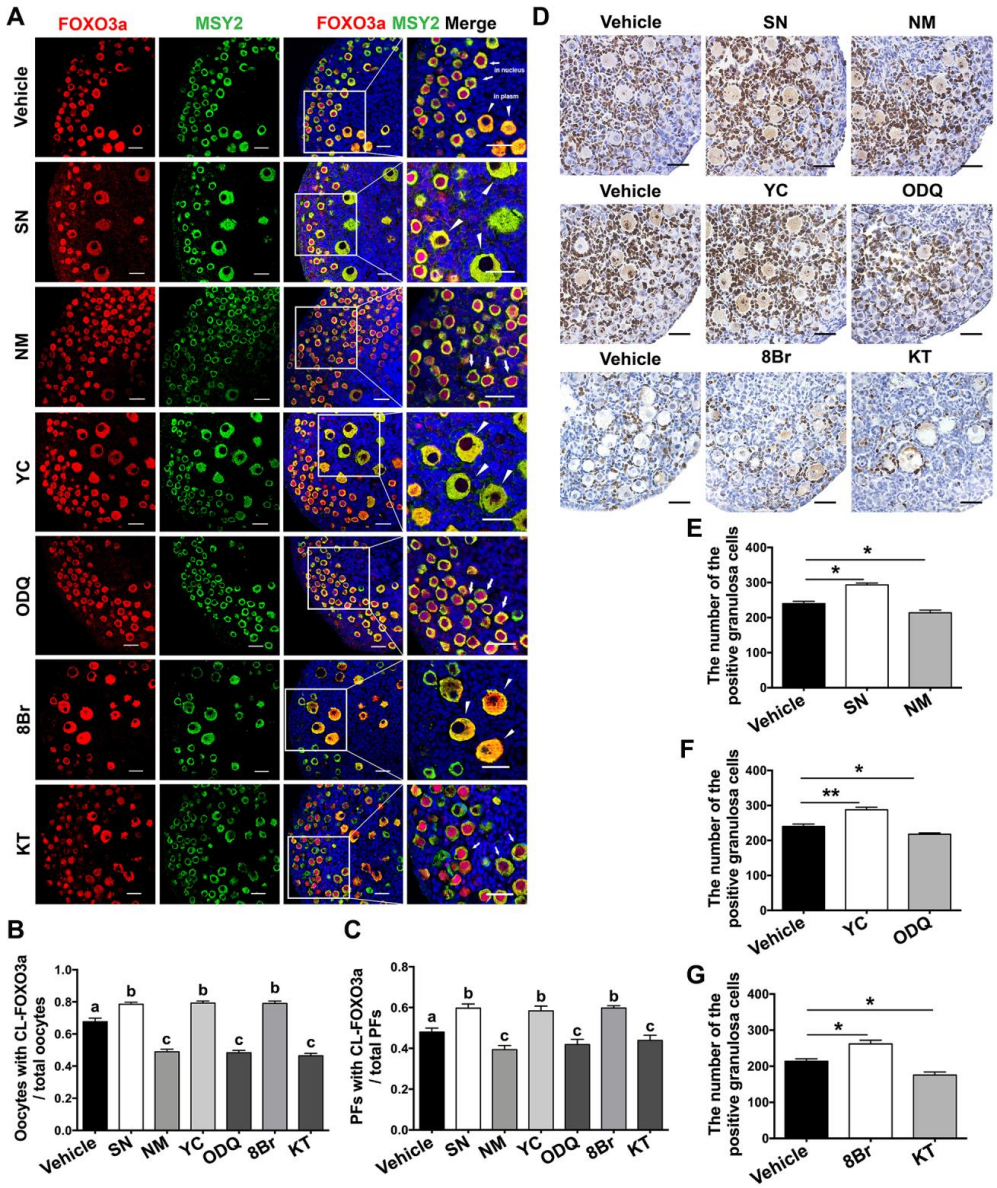
**The eNOS/cGMP/PKG pathway promoted oocyte FOXO3a translocation and granulosa cell proliferation during PF activation.** (**A**) Immunofluorescence staining of FOXO3a was performed in ovaries treated with the vehicle, SN (100 μM), NM (1 mM), YC (10 μM), ODQ (1 μM), 8Br (10 μM) or KT (1 μM) (n=6). Arrows indicate PFs and triangles indicate GFs. Scale bar, 40 μm. (**B**) The number of oocytes with cytoplasmic localization of FOXO3a (CL-FOXO3a)/the total number of oocytes was analyzed (n=8). (**C**) The number of PFs with CL-FOXO3a/the total number of PFs was analyzed in each group (n=8). (**D**) Ki67 staining was carried out in ovaries treated with the vehicle, SN (100 μM), NM (1 mM), YC (10 μM), ODQ (1 μM), 8Br (10 μM) or KT (1 μM) for six days. Scale bar, 40 μm. (**E**–**G**) Ki67-positive somatic cells in follicles were counted in all the groups (n=6). Different letters denote statistical significance at *p* < 0.01. * and ** denote statistical significance at *p* < 0.05 and *p* < 0.01, respectively.

The transcription factor FOXO3a, a direct substrate of AKT, suppresses PF activation when it is localized in the nuclei of PF oocytes [1]. To explore the possible mechanisms by which the eNOS/cGMP/PKG pathway activated PFs, we assessed the translocation of FOXO3a in oocytes and the proliferation of granulosa cells. Immunofluorescence assays indicated that FOXO3a was exported from the nucleus to the cytoplasm and colocalized with the germline marker Y box protein 2 (MSY2) in most oocytes in the SN, YC and 8Br groups. On the contrary, FOXO3a remained in the nuclei of oocytes in the NM, ODQ or KT groups (Figure 4A, 4B). Consistently, the proportion of PFs in which FOXO3a was localized in the cytoplasm increased upon treatment with SN, YC or 8Br, but decreased upon treatment with NM, ODQ or KT (Figure 4A, 4C).

In addition, Ki67 staining was performed to evaluate cell proliferation in the treated ovaries. The percentage of Ki67-positive granulosa cells increased significantly after SN, YC or 8Br treatment, but decreased after NM, ODQ or KT treatment compared with vehicle treatment (Figure 4D–4G). These results indicated that the eNOS/cGMP/PKG pathway promotes the nuclear export of FOXO3a in oocytes and the proliferation of granulosa cells during PF activation, suggesting that the PI3K/AKT/FOXO3a pathway is stimulated during eNOS/cGMP/PKG-induced follicle activation.

### The eNOS/cGMP/PKG pathway activated PFs via mTOR

**Figure 5:**
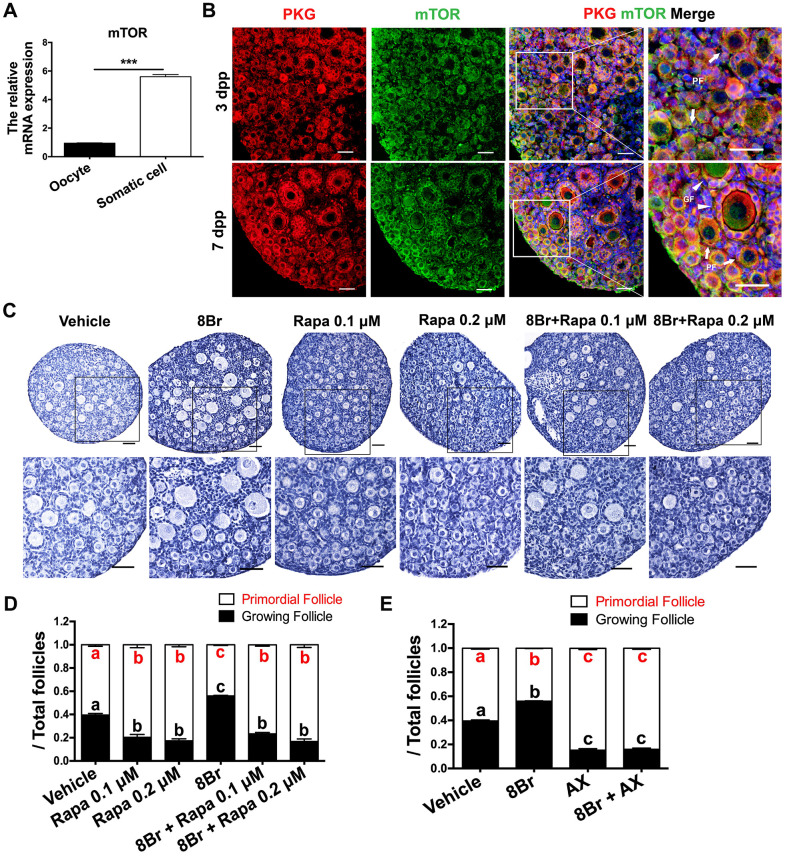
**The eNOS/cGMP/PKG pathway activated PFs via mTOR in preGCs.** (**A**) The relative mRNA levels of mTOR in oocytes and somatic cells isolated from ovaries at 3 dpp. *** denotes statistical significance at *p* < 0.001. (**B**) The colocalization of mTOR and PKG was detected via immunofluorescence in neonatal ovaries at 3 and 7 dpp. Arrows indicate PFs and triangles indicate GFs. Scale bar, 40 μm. Ovaries at 1 dpp were treated with the vehicle, 8Br (10 μM), the mTOR inhibitor rapamycin (Rapa) or 8Br + Rapa for six days (n=6). (**C**) The ovarian morphology and 9 (**D**) the numbers of PFs and GFs/the total number of follicles were analyzed. Scale bar, 40 μm. (**E**) Ovaries at 1 dpp were treated with the vehicle, 8Br (10 μM), the KIT inhibitor AX (5 μM) or 8Br + AX for six days (n=6). The numbers of PFs and GFs/the total number of follicles were analyzed. Different letters with the same color denote statistical significance at *p* < 0.001 (Red letters represent the proportions of PFs, while black letters represent the proportions of GFs).

It has been well described that mTOR in preGCs promotes PF activation by inducing the KIT/ PI3K/AKT/FOXO3a pathway [10]. Thus, we investigated whether mTOR was involved in the eNOS/cGMP/PKG-induced activation of PFs. A qRT-PCR assay revealed that mTOR was overwhelmingly expressed in the somatic cells of the ovaries at 3 dpp (Figure 5A). In an immunofluorescence assay, mTOR colocalized with PKG in the preGCs of PFs (Figure 5B). When the ovaries were treated with the cGMP analog 8Br and the mTOR inhibitor rapamycin simultaneously, both the ovarian morphology and the follicle count indicated that rapamycin prevented 8Br from activating PFs (Figure 5C, 5D, Supplementary Figure 3A–3E). Likewise, the KIT inhibitor Axitnib (AX) reversed the effects of 8Br on the follicle count (Figure 5E, Supplementary Figure 3F–3J). These results demonstrated that the eNOS/cGMP/PKG pathway activates PFs via mTOR in preGCs and its downstream KIT.

### The eNOS/cGMP/PKG pathway stabilized mTOR protein

**Figure 6:**
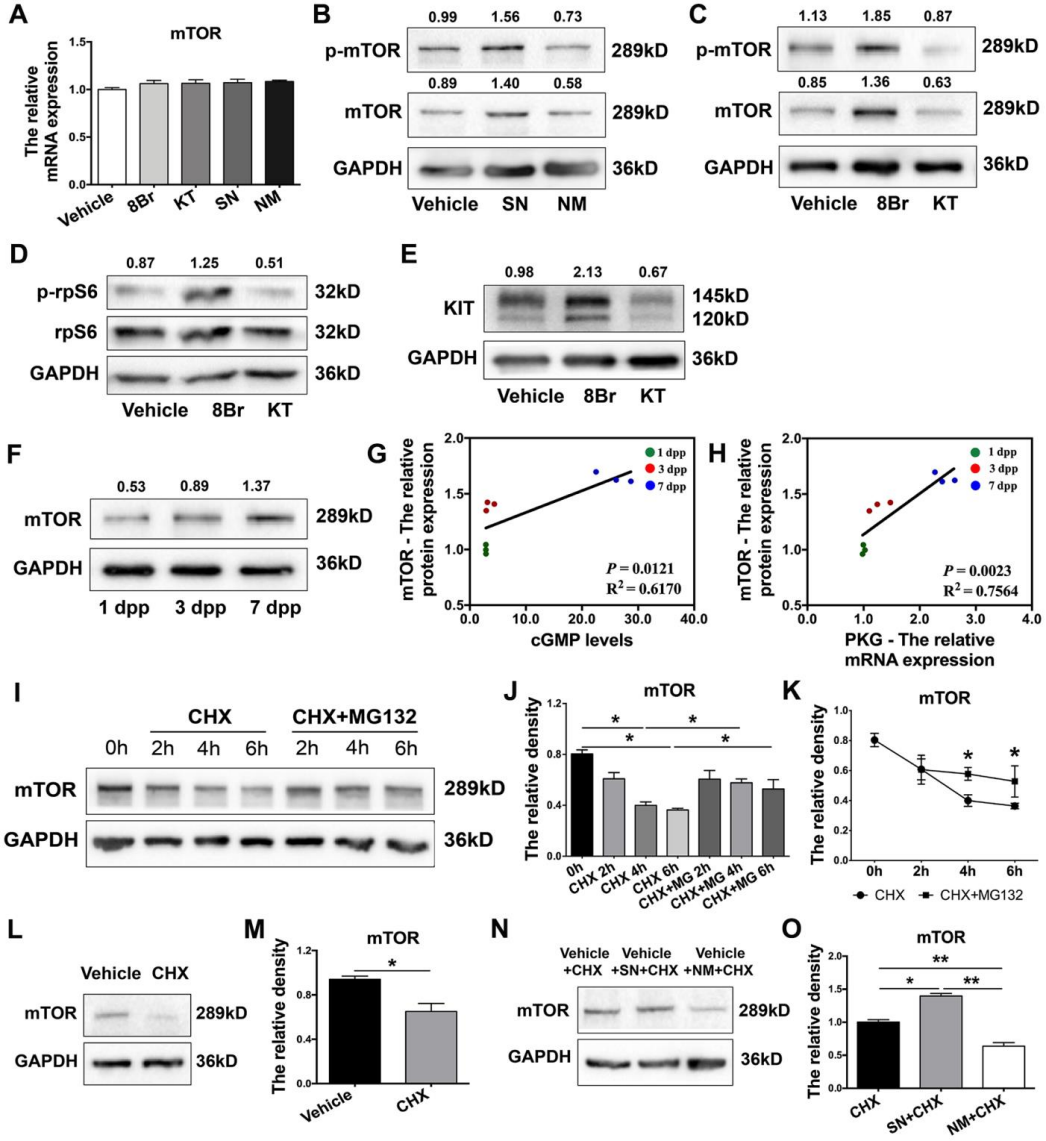
**The eNOS/cGMP/PKG pathway stabilized mTOR protein.** (**A**) The relative mRNA levels of mTOR in ovaries treated with the vehicle, SN, NM, 8Br or KT. (**B**, **C**) Western blotting analysis of mTOR protein levels in ovaries treated with the vehicle, SN, NM, 8Br or KT. (**D***,* **E**) Western blotting analysis of p-rpS6, rpS6 and KIT protein levels in ovaries treated with the vehicle, 8Br or KT. (**F**) Western blotting analysis of mTOR protein levels in ovaries from 1-7 dpp. (**G**) The correlation between the levels of cGMP and the protein levels of mTOR. (**H**) The correlation between the mRNA levels of PKG and the protein levels of mTOR. (**I***–***K**) Western blotting analysis and the relative density of mTOR protein levels in HEK293T cells treated with cycloheximide (CHX, 40 μM) or CHX + MG132 (10 μM). (**L**, **M**) Western blotting analysis and the relative density of mTOR protein levels in ovaries treated with CHX for 4 h. (**N**, **O**) Western blotting analysis and the relative density of mTOR protein levels in ovaries treated with CHX, CHX + SN or CHX + NM for 4 h. * and ** denote statistical significance at *p* < 0.05 and *p* < 0.01, respectively.

Next, we used various activators and inhibitors to further investigate how the eNOS/cGMP/PKG pathway regulated mTOR. We found that mTOR mRNA levels were not altered by the activation or inhibition of the eNOS/cGMP/PKG pathway in neonatal ovaries (Figure 6A). In contrast, the protein levels of mTOR were significantly increased by the agonists and reduced by the inhibitors of the eNOS/cGMP/PKG pathway (Figure 6B, 6C). The levels of phosphorylated (p)-mTOR and its downstream effectors phosphorylated ribosomal protein S6 (p-rpS6) and KIT were also elevated by the agonists and reduced by the inhibitors (Figure 6B–6E). Moreover, mTOR protein levels in the ovaries increased continuously from 1 dpp to 7 dpp (Figure 6F). The levels of mTOR protein correlated positively with the levels of cGMP and PKG mRNA (Figure 6G, 6H). These results demonstrated that the eNOS/cGMP/PKG pathway upregulates mTOR protein.

Subsequently, we measured the disappearance of mTOR protein in HEK293T cells treated with the translation inhibitor cycloheximide. The degradation of mTOR protein became significant at 4 h, but this effect was completely reversed by the proteasome inhibitor MG132 (Figure 6I–6K). In neonatal ovaries, mTOR protein was also significantly degraded 4 h after cycloheximide treatment (Figure 6L, 6M), indicating that mTOR can be rapidly degraded in a proteasome-dependent manner. Notably, the stability of mTOR protein in cycloheximide-treated neonatal ovaries was enhanced upon treatment with the NO donor SN and reduced upon treatment with the NOS inhibitor MN (Figure 6N, 6O). These results demonstrated that the eNOS/cGMP/PKG pathway stabilizes mTOR protein in neonatal ovaries.

### FBXW7 destabilized mTOR protein in a ubiquitin/proteasome-dependent manner

**Figure 7:**
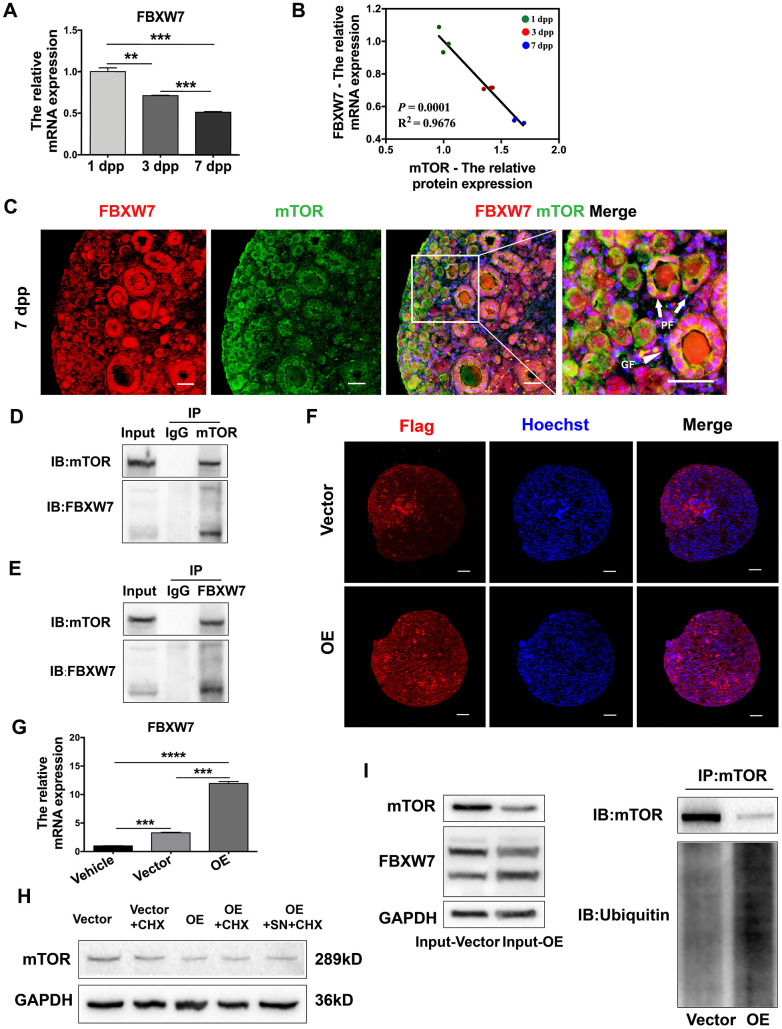
**FBXW7 destabilized mTOR protein in a ubiquitin/proteasome-dependent manner.** (**A**) The relative mRNA levels of FBXW7 in ovaries from 1-7 dpp. (**B**) The correlation between the mRNA levels of FBXW7 and the protein levels of mTOR. (**C**) Immunofluorescence analysis displaying the colocalization of mTOR and FBXW7 in neonatal ovaries. Arrows indicate PFs and triangles indicate GFs. Scale bar, 40 μm. (**D**) Immunoblotting analysis of Co-IP against mTOR. (**E**) Immunoblotting analysis of Co-IP against FBXW7. (**F**) Six days after transfection, immunofluorescence staining for Flag was performed in the empty vector- and FBXW7 overexpression (OE) vector-treated ovaries. Scale bar, 40 μm. (**G**) Three days after transfection, the relative mRNA levels of FBXW7 were detected in the empty vector and OE vector groups. (**H**) Western blotting analysis of mTOR protein levels in ovaries treated for 4 h with CHX or SN + CHX three days after transfection. (**I**) Immunoblotting analysis of Co-IP against mTOR in ovaries three days after transfection. ** and *** denote statistical significance at *p* < 0.01 and *p* < 0.001, respectively.

FBXW7 is a major E3 ubiquitin ligase that promotes proteasomal mTOR protein degradation [36, 38]. Thus, we examined whether FBXW7-induced mTOR ubiquitination was responsible for the destabilization of mTOR protein in neonatal ovaries. The mRNA levels of FBXW7 in neonatal ovaries declined gradually from 1 dpp to 7 dpp (Figure 7A), and correlated negatively with mTOR protein levels (Figure 7B). Immunofluorescence staining revealed that FBXW7 and mTOR colocalized in the cytoplasm of preGCs in PFs (Figure 7C). More significantly, a co-immunoprecipitation (Co-IP) assay indicated that mTOR and FBXW7 bound to each other in 3 dpp ovaries (Figure 7D, 7E), suggesting that these proteins bind directly to one another in preGCs.

Next, we overexpressed FBXW7 in 1 dpp ovaries by injecting them with a Flag-tagged FBXW7 overexpression lentivirus. FBXW7 mRNA levels increased significantly 72 h after transfection (Figure 7G), and Flag could be stained in the whole ovaries six days after transfection (Figure 7F), indicating the high transfection efficiency of the FBXW7 overexpression vector in the neonatal ovaries. The overexpression of FBXW7 reduced the protein levels of mTOR, and destabilized mTOR protein as detected in a cycloheximide chase assay (Figure 7H). A Co-IP assay demonstrated that ubiquitin-modified mTOR levels were significantly greater in FBXW7 overexpression vector-treated ovaries than in empty vector-treated ovaries (Figure 7I). These results indicated that FBXW7 reduced the stability of mTOR protein in a ubiquitin/proteasome-dependent manner in preGCs from neonatal ovaries.

### The eNOS/cGMP/PKG pathway activated PFs by inhibiting FBXW7-induced mTOR ubiquitination

**Figure 8:**
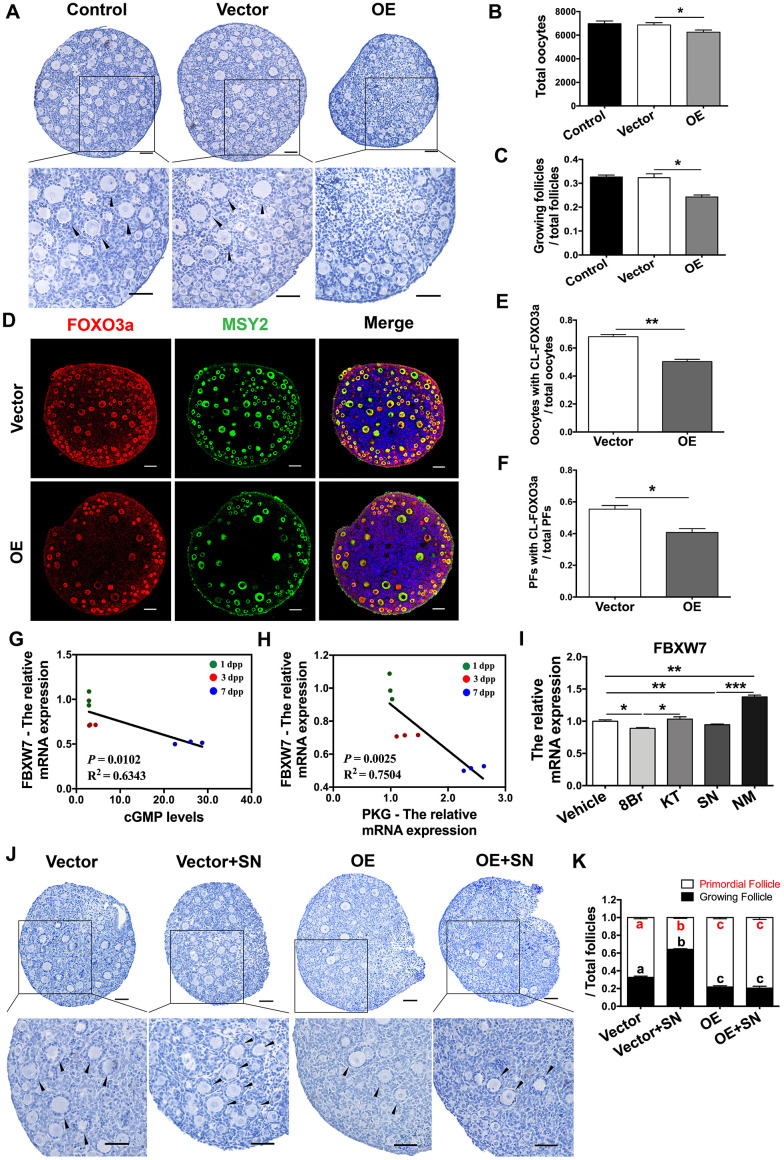
**The eNOS/cGMP/PKG pathway activated PFs by inhibiting FBXW7-induced mTOR ubiquitination.** (**A**–**C**) Six days after transfection, (**A**) the ovarian morphology, (**B**) the total number of oocytes and (**C**) the numbers of PFs and GFs/the total number of follicles were analyzed (n=8). Scale bar, 40 μm. (**D**) Immunofluorescence staining of FOXO3a in the empty vector and OE vector groups. Scale bar, 40 μm. (**E**) The number of oocytes with CL-FOXO3a/the total number of oocytes was analyzed in each group (n=8). (**F**) The number of PFs with CL-FOXO3a/the total number of PFs was analyzed in each group (n=8). (**G**) The negative correlation between the relative mRNA levels of FBXW7 and PKG. (**H**) The negative correlation between the relative mRNA levels of FBXW7 and the levels of cGMP. (**I**) The relative mRNA levels of FBXW7 in ovaries treated with the vehicle, SN, NM, 8Br or KT. *, ** and *** denote statistical significance at *p* < 0.05, *p* < 0.01 and *p* < 0.001, respectively. (**J, K**) After treatment with the empty vector, empty vector + SN, OE vector or OE vector + SN for six days, (**J**) the ovarian morphology, and (**K**) the numbers of PFs and GFs/the total number of follicles were analyzed (n=8). Scale bar, 40 μm. Different letters with the same color denote statistical significance at *p* < 0.01 (Red letters represent the proportions of PFs, while black letters represent the proportions of GFs).

The above results indicated that the stability of mTOR protein was reduced by FBXW7 but enhanced by the eNOS/cGMP/PKG pathway. Therefore, we investigated whether FBXW7 was also involved in PF activation or regulated by the eNOS/cGMP/PKG pathway. In FBXW7-overexpressing neonatal ovaries, the proportions of PFs were significantly greater and the proportions of GFs were significantly lower than those in the control and empty vector group (Figure 8A–8C). Consistently, upon FBXW7 overexpression, FOXO3a remained localized in the nucleus in most oocytes and PFs, and the export of FOXO3a from the nucleus was significantly inhibited (Figure 8D–8F). These data indicated that FBXW7 suppresses PF activation.

Interestingly, the levels of FBXW7 mRNA correlated negatively with the levels of cGMP and PKG mRNA in neonatal ovaries (Figure 8G, 8H). Agonists of the eNOS/cGMP/PKG pathway significantly downregulated FBXW7 in the ovaries, while inhibitors of the eNOS/cGMP/PKG pathway upregulated FBXW7 (Figure 8I). Additionally, FBXW7 overexpression prevented the NO donor SN from activating PFs and stabilizing mTOR protein (Figure 8J, 8K, and Figure 7H). These results demonstrated that the eNOS/cGMP/PKG pathway activates PFs by suppressing the FBXW7-induced ubiquitination of mTOR in preGCs.

## DISCUSSION

The well-tuned recruitment of PFs is needed for a normal female reproductive life-span. In the present study, we found that the eNOS/cGMP/PKG pathway activated PFs by stabilizing mTOR protein, promoting granulosa cell proliferation and inducing FOXO3a translocation in oocytes. The ubiquitin E3 ligase FBXW7 induced mTOR ubiquitination/degradation in preGCs, suppressed PF activation, prevented eNOS from activating PFs and stabilizing mTOR, and was downregulated by the eNOS/cGMP/PKG pathway. These results demonstrated that the eNOS/cGMP/PKG pathway activates PFs by inhibiting FBXW7-induced mTOR ubiquitination in mice.

eNOS is essential for ovarian development in mammalian females. In adult rats, eNOS is expressed in oocytes, theca cells and mural granulosa cells of preantral and antral follicles, and its expression can be promoted by gonadotropins [24, 25]. Mice deficient in eNOS display a prolonged estrous cycle, severely impaired oocyte meiotic maturation and ovulation (even after superovulation), and a significant reduced litter size [26, 44]. NOS substrate injection was found to increase the proportion of primary and antral follicles and promote the phosphorylation of AKT/FOXO3a in rats [28]. Here we demonstrated that eNOS induced PF activation, oocyte growth, oocyte FOXO3a nuclear export and granulosa cell proliferation in mice. Although the deletion of the other two isoforms of NOS (iNOS and nNOS) was reported to prolong estrous cyclicity in females [26, 45], we found that iNOS and nNOS were barely expressed in neonatal ovaries, and inhibiting them did not alter follicle activation. These results suggested that eNOS, but not iNOS or nNOS, activates PFs in mice.

Of note, eNOS was expressed in both oocytes and preGCs in PFs. It is possible that NO synthesized by eNOS in oocytes diffuses to the neighboring preGCs, where it can bind to sGC to activate cGMP/PKG. However, it is also possible that NO produced by preGCs binds autonomously to sGC to activate cGMP/PKG in preGCs. Germ cell-specific and preGC-specific eNOS knockout mice could be used to clarify the dominant source of NO during PF activation.

Serine/threonine-specific PKG, which is stimulated by cGMP, has been shown to suppress hormone release, enhance glucose uptake and inhibit apoptosis in mammalian granulosa cells [30–32]. We investigated whether the cGMP/PKG pathway contributed to early follicle development. Our data indicated that sGC and PKG were predominantly located in preGCs, and that the cGMP/PKG pathway was necessary for eNOS-induced PF activation. Natriuretic peptide receptor 2, the major membrane guanylyl cyclase, can produce cGMP in the ovaries. However, the mutation of natriuretic peptide receptor 2 did not influence early folliculogenesis in mice [29, 45], illustrating that the natriuretic peptide receptor/cGMP system is not involved in PF activation. In addition, cGMP maintains oocyte meiotic arrest and cardiac contractility by inhibiting cyclic adenosine monophosphate (cAMP) phosphodiesterase [46, 47]. We found that cAMP also activated PFs. Interestingly, disrupting cAMP did not alter cGMP activity, but inhibiting cGMP generation reversed the effects of cAMP (Supplementary Figure 4), implying that cAMP may also be an upstream regulator of cGMP during PF activation. Overall, our results demonstrated that NO generated by eNOS activates cGMP/PKG in preGCs, ultimately activating PFs.

As an atypical serine/threonine kinase, mTOR governs cell growth and metabolism through multiprotein complexes in response to various nutrients and growth factors [48]. In early ovarian development, mTOR signaling in preGCs triggers PF activation. Previous studies have shown that mTOR promotes the differentiation of preGCs, and induces KIT in oocytes by upregulating preGC KITL, and activates the downstream PI3K/AKT/FOXO3a pathway, which consequently awakens dormant oocytes and PFs [14–19]. Due to its functions in PF activation, mTOR has become a key target in the treatment of POI. Nutrition, oxygen and growth factors are known to be upstream regulators of mTOR signaling [49], but the specific upstream regulators of mTOR in preGCs have not been delineated. We found that mTOR colocalized with PKG in preGCs, and the mTOR inhibitor rapamycin prevented the eNOS/cGMP/PKG pathway from activating PFs. The eNOS/cGMP/PKG pathway stabilized mTOR protein and consequently increased the levels of p-mTOR, p-rpS6 and KIT in neonatal ovaries. Thus, the eNOS/cGMP/PKG pathway activates PFs by stabilizing mTOR protein in preGCs.

Next, we explored the mechanisms behind mTOR stabilization in neonatal ovaries. FBXW7, the substrate recognition subunit of the E3 ubiquitin ligase complex SKP1-cullin-F-box, facilitates ubiquitin-dependent degradation of various oncoproteins [35]. FBXW7 promotes mTOR ubiquitination/degradation in cancer cells and gliocytes, while FBXW7 deficiency increases the sensitivity of a variety of human tumors to rapamycin [33–36, 50]. Interestingly, FBXW7 only binds to the mTOR complex mTORC1 [40], which is also the only mTOR complex involved in PF activation [10]. In contrast, FBXW7 does not promote the degradation of mTORC2. We found that FBXW7 levels correlated negatively with mTOR protein levels, and that FBXW7 bound directly to mTOR in preGCs. FBXW7 overexpression significantly promoted mTOR ubiquitination/degradation, inhibited PF activation and prevented the NO donor from activating PFs and stabilizing mTOR. The eNOS/cGMP/PKG pathway downregulated FBXW7 in the ovaries, demonstrating that the eNOS/cGMP/PKG pathway stabilizes mTOR protein and activates PFs by reducing FBXW7 mRNA expression.

The eNOS/cGMP/PKG pathway seems to downregulate FBXW7 indirectly, since eNOS, cGMP, PKG and their direct downstream effectors are not known to bind to FBXW7. Of note, miR-223 and a number of other microRNAs directly silence FBXW7 [51, 52], and nuclear factor κB (NFκB) and Notch cooperatively inhibit FBXW7 by directly activating the transcription of miR-223 [53]. The NO/cGMP/PKG pathway is an upstream activator of both NFκB and Notch signaling [54, 55]. Therefore, we speculate that the NO/cGMP/PKG pathway may inhibit FBXW7 expression by activating NFκB/Notch/miR-223 in preGCs.

We also found that FBXW7 expression slightly reduced the total oocyte numbers. FBXW7 is known to promote apoptosis by degrading several anti-apoptotic factors, including the Bcl-2 family member myeloid cell leukemia sequence 1 (Mcl-1) [56, 57]. Mcl-1 is expressed in preantral follicle oocytes, and is required for oocyte survival [58, 59]. Thus, FBXW7 overexpression in neonatal ovaries may have induced the ubiquitination/degradation of anti-apoptotic factors such as Mcl-1, ultimately promoting oocyte apoptosis. Therefore, maintaining the proper quantity and activity of FBXW7 is essential for both oocyte survival and PF activation.

**Figure 9:**
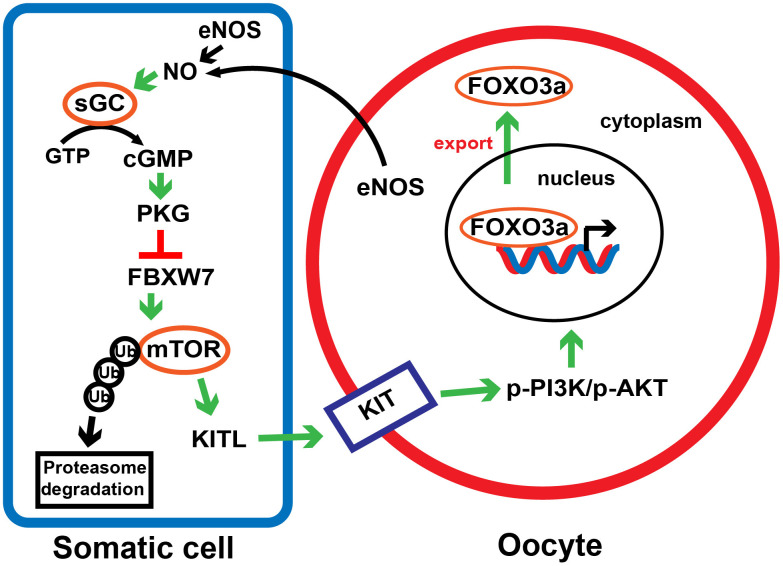
**A proposed model depicting the involvement of the eNOS/cGMP/PKG pathway in PF activation.** In response to NO synthesized by eNOS in oocytes and preGCs, cGMP and PKG are activated and downregulates FBXW7 in preGCs, thus diminishing FBXW7-induced ubiquitination/degradation of mTOR. This process triggers the transduction of KITL/KIT/PI3K/AKT/FOXO3a signaling, links the communication between preGCs and oocytes, and consequently awakens dormant PFs.

In conclusion, we demonstrated that the eNOS/cGMP/PKG pathway activates PFs by inhibiting the FBXW7-induced ubiquitination of mTOR in mice (Figure 9). The observations have deepened our understanding of early follicle development, and may provide novel targets for *in vitro* activation and POI treatment.

## MATERIALS AND METHODS

### Animals

The ICR mice used in this study were purchased from Beijing Vital Laboratory Animal Technology Co. (China). Eight-week-old female mice were mated with fertile males overnight to induce pregnancy. All mice were maintained on a 12/12 h light/dark cycle at 26° C, and were allowed free access to food and water. All the experiments and animal procedures were conducted in accordance with the guidelines of the Animal Experiments and Experimental Animal Management Committee of Capital Medical University. The study protocol was approved by the Animal Experiments and Experimental Animal Welfare Committee of Capital Medical University (permit number: AEEI-2017-021).

### Neonatal ovaries and cell culture

Ovaries were retrieved from neonatal mice and cultured in serum-free Dulbecco’s modified Eagle’s medium (DMEM)/Ham’s F12 nutrient mixture (Gibco, USA) with 100 IU/mL penicillin-streptomycin (KGY0023, Keygen Biotech, China). The HEK293T cell line was purchased from Keygen Biotech (KG405) and cultured in DMEM (Gibco) containing 10% fetal bovine serum (SE100-B, VISTECH, New Zealand) and 100 IU/mL penicillin-streptomycin (Keygen Biotech). The ovaries and cells were maintained at 37° C with 5% CO_2_ and saturated humidity in a humidified incubator.

### Reagents

The NO donor SNAP (‘SN’, N3398), the sGC agonist YC-1 (‘YC’, Y102), the sGCs inhibitor ODQ (O3636), the cGMP analog 8-Br-cGMP (‘8Br’, B1381), the mTOR inhibitor rapamycin (R8781), the cAMP donor dbcAMP (D0260) and the adenylyl cyclase inhibitor MDL-12,330A (‘MDL’, M182) were all purchased from Sigma-Aldrich (USA). The PKG inhibitor KT5823 (‘KT’, S1691), the NOS inhibitor L-NMMA (‘NM’, S0011), the eNOS inhibitor L-NAME (‘NA’, S0006), the iNOS inhibitor SMT (‘SM’, S0008), the nNOS inhibitor spermidine (‘SP’, S0010) and the proteasome inhibitor MG-132 (‘MG132’, S1748) were purchased from Beyotime (China). The KIT inhibitor Axitnib (‘AX’, S1005) was purchased from Selleck (China). The protein synthesis inhibitor cycloheximide (HY-12320) was purchased from MedChemExpress (USA).

### Follicle counting

The numbers of follicles and oocytes were counted according to a generally accepted approach [10]. Briefly, the embedded ovaries were serially sliced into 5-μm sections, and the numbers of oocytes and follicles were counted in every fifth section. The total numbers of oocytes and follicles in each ovary were then multiplied by five, and six ovaries from each group were counted.

The stages of the follicles were classified according to accepted published definitions [60]: 1) PF: an oocyte surrounded by a single layer of flattened preGCs; 2) primary follicle: an oocyte surrounded by a single layer of cuboidal granulosa cells; 3) secondary follicle: an oocyte surrounded by two or more layers of cuboidal granulosa cells without an antrum. Primary and secondary follicles were classified as GFs. The number of proliferating granulosa cells and the proportions of oocytes or PFs with cytoplasmic localization of FOXO3a (CL-FOXO3a) were analyzed in three serial sections around the largest cross-section of each ovary, and were averaged.

### Separation of oocytes and somatic cells from ovarian cells

The ovaries at 3 dpp were collected and incubated with 200 μL of a 0.125% trypsin solution (SH30042, Hyclone) at 37° C for 5-10 min and then at 4° C for 30 min for further digestion. When there were no longer any noticeable lumps, the digestion reaction was terminated with fetal bovine serum. The cells were than washed, resuspended in 500 μL of DMEM/F12 with modified Insulin-Transferrin-Sodium Selenite (I1884, Sigma-Aldrich), and cultured for 12 h. After being thus cultured, the oocytes remained suspended in the medium, while the granulosa cells adhered to the culture dishes. The plates were mildly shaken to flush out any loosely adhering oocytes and the supernatants were centrifuged to harvest the oocytes. After being washed and digested, the attached somatic cells on the plates were harvested.

### RNA extraction and qRT-PCR

Total RNA was extracted from 10 ovaries from each group using TRIZOL Reagent (Invitrogen, USA), and was reverse-transcribed with a FastQuant RT Kit (with gDNase) (KR106, TIANGEN, China) according to the manufacturer’s protocol. Then, qRT-PCR was conducted using SuperReal PreMix Plus (FP205, TIANGEN) on a CFX96 Touch qPCR Detection System (Bio-Rad, USA). The expression of each gene was normalized to that of β-actin, and then the relative fold-change was calculated using the 2^−ΔΔCt^ method. The primers are listed in Supplementary Table 1.

### Immunohistochemistry and immunofluorescence

Neonatal ovaries were fixed in 4% polyformaldehyde at 4° C overnight, dehydrated in serial ethanol dilutions, hyalinized in xylene, embedded in paraffin and cut into 5-μm-thick sections. After the samples were dewaxed and rehydrated, antigen retrieval and serum blocking were performed. For immunohistochemistry, the ovary sections were incubated with primary antibodies at 4° C overnight. All the primary antibodies and their dilutions are listed in Supplementary Table 2. The sections were washed, incubated with horseradish peroxidase-labeled goat IgG (SP-9002, ZSBIO, China) for 20 min at room temperature, and then treated with DAB reagent (ZLI-9018, ZSBIO). The sections were counterstained with hematoxylin, dehydrated, coverslipped and imaged using a Nikon Eclipse E100 microscope (Nikon, Japan).

For immunofluorescence, after antigen retrieval, the ovary sections were blocked with 10% normal donkey serum (ab7475, Abcam, UK) for 1 h at room temperature and incubated with primary antibodies overnight at 4° C (Supplementary Table 2). After being incubated with Alexa Fluor 488- or Alexa Fluor 555-conjugated secondary antibodies (1:200, A31572, A21202, A32814, Invitrogen) for 1 h at 37° C, the sections were stained with Hoechst 33342 (1:100, B2261, Sigma-Aldrich) for 1 min, and sealed in anti-fade fluorescence mounting medium (S2100, Solarbio, China) with coverslips. The fluorescence images were examined and photographed using a Leica SP8 laser scanning confocal microscope (Leica, Germany) or a Zeiss AX4 fluorescence microscope (Zeiss, Germany).

### Radioimmunoassay

Neonatal ovaries were dissolved in 100 μL of 1 M HCl and placed on ice for 10 min. The sample solutions were then frozen in liquid nitrogen and stored at -80° C. For the radioimmunoassay, the solutions were thawed, centrifuged at 12,000 x g for 10 min and dried at 60° C overnight. Then, cGMP levels were measured with a cGMP [125I] radioimmunoassay kit (IZOTOP, Hungary). The protein concentrations were measured with a Pierce Rapid Gold BCA Protein Assay Kit (Thermo Scientific, USA).

### Western blotting

The total proteins from each sample were separated by electrophoresis on 8% sodium dodecyl sulfate-polyacrylamide gels and transferred to polyvinylidene fluoride membranes (IPVH00010, Merck Millipore, USA). After being blocked with 5% skim milk for 1 h at room temperature, the membranes were incubated with primary antibodies at 4° C overnight (Supplementary Table 2). Then, the membranes were incubated with peroxidase-conjugated secondary antibodies (1:5000, E-AB-1003, Elabscience, China) for 1 h at room temperature. The proteins on the membranes were visualized with Immobilon Western HRP Substrate (WBKLS0500, Merck Millipore) and scanned on a SuperSignal chemiluminescent detection system (BioRad, USA).

### Co-IP

Ovaries or cells were lysed by IP lysis buffer (87787, Thermo Scientific) containing a serine/cysteine protease inhibitor (1 mM phenylmethanesulfonyl fluoride; P7626, Merck Millipore) and a cysteine protease inhibitor (5 mM N-Ethylmaleimide; HY-D0843, MedChemExpress). Co-IP was carried out using a Dynabeads® Protein G Immunoprecipitation Kit (10007D, Invitrogen). Briefly,10 μg of the primary antibodies were incubated with 1.5 mg of Dynabeads at 4° C for 1 h with rotation. Then, 1 mg of the protein lysates was added to the Dynabeads-antibody complex and incubated at 4° C overnight with rotation. Finally, the proteins were eluted, denatured and subjected to Western blotting.

### Lentiviral vector construction and injection

Mouse Flag-tagged-*FBXW7* cDNA was cloned into a pCDH Expression Lentivector. The expression construct was co-transfected with psPAX2 and pMd2.G plasmids into HEK293T cells to produce lentiviral vectors. The viral supernatants were collected via ultracentrifugation. As previously reported [61, 62], we used a glass needle to inject the lentiviral vectors into the cortex of the neonatal ovaries at 1 dpp, selecting at least three parts. For each ovary, at least 0.3 μL of the lentiviral suspension (1×10^8^ IU/mL) was injected.

### Statistical analysis

All experiments were repeated at least three times, and all data are reported as the mean ± standard error of the mean. Pearson correlation analysis was conducted in SPSS 12.0 (IBM, USA), while other data were analyzed using a *t*-test or analysis of variance (GraphPad Prism 6.0, GraphPad Software, USA). Differences were considered significant at *p* < 0.05.

## Supplementary Material

Supplementary Figures

Supplementary Tables
