# Supplementary material for: The endothelial nitric oxide synthase/cyclic guanosine monophosphate/protein kinase G pathway activates primordial follicles
Source: Aging (Albany NY). 2020 Dec 3;13(1):1096–119. doi: 10.18632/aging.202235 (PMC7835019; doi:10.18632/aging.202235)
Supplement: Supplementary Figures [file aging-13-202235-s001.pdf]

SUPPLEMENTARY FIGURES

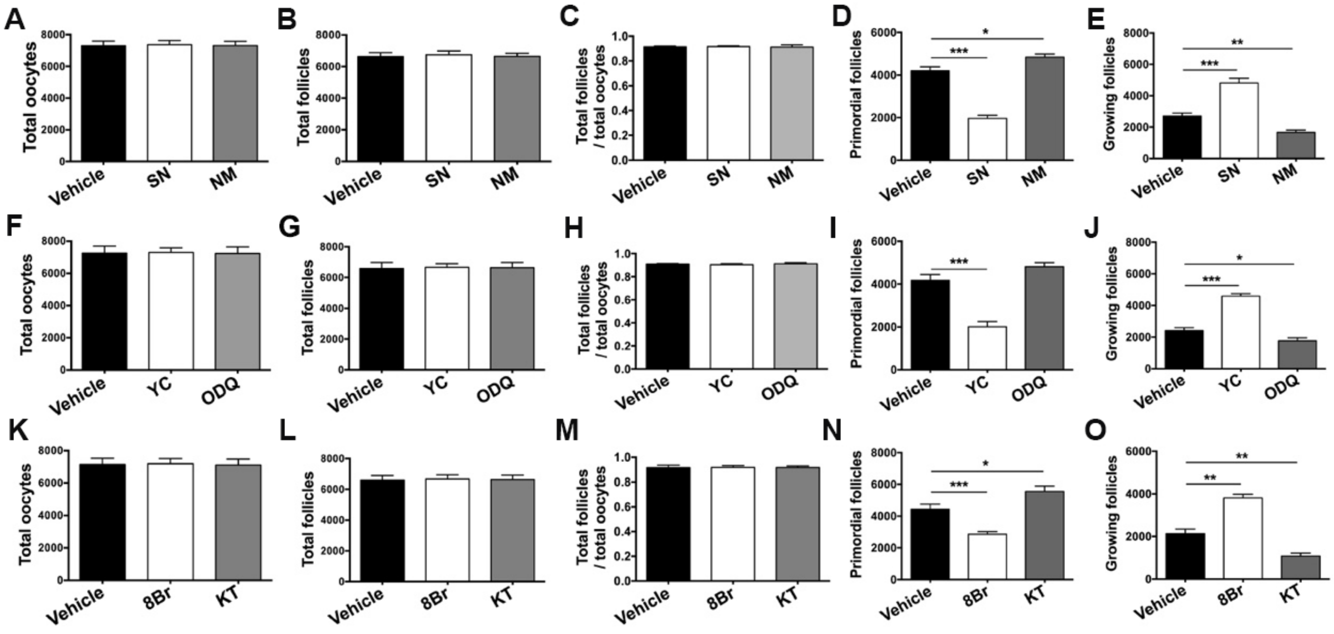

Supplementary Figure 1. The numbers of oocytes, follicles, PFs and GFs after stimulating or inhibiting the eNOS/cGMP/PKG pathway. (**A**) The total number of oocytes, (**B**) the total number of follicles, (**C**) the total number of follicles/the total number of oocytes, (**D**) the number of PFs and (**E**) the number of GFs were counted in ovaries treated with the vehicle, SN (100 μM) or NM (1 mM) for six days (n=6). (**F**) The total number of oocytes, (**G**) the total number of follicles, (**H**) the total number of follicles/the total number of oocytes, (**I**) the number of PFs and (**J**) the number of GFs were counted in ovaries treated with the vehicle, YC (10 μM) or ODQ (1 μM) for six days (n=6). (**K**) The total number of oocytes, (**L**) the total number of follicles, (**M**) the total number of follicles/the total number of oocytes, (**N**) the number of PFs and (**O**) the number of GFs were counted in ovaries treated with the vehicle, 8Br (10 μM) or KT (1 μM) for six days (n=6). *, ** and *** denote statistical significance at $p < 0.05$, $p < 0.01$ and $p < 0.001$, respectively.

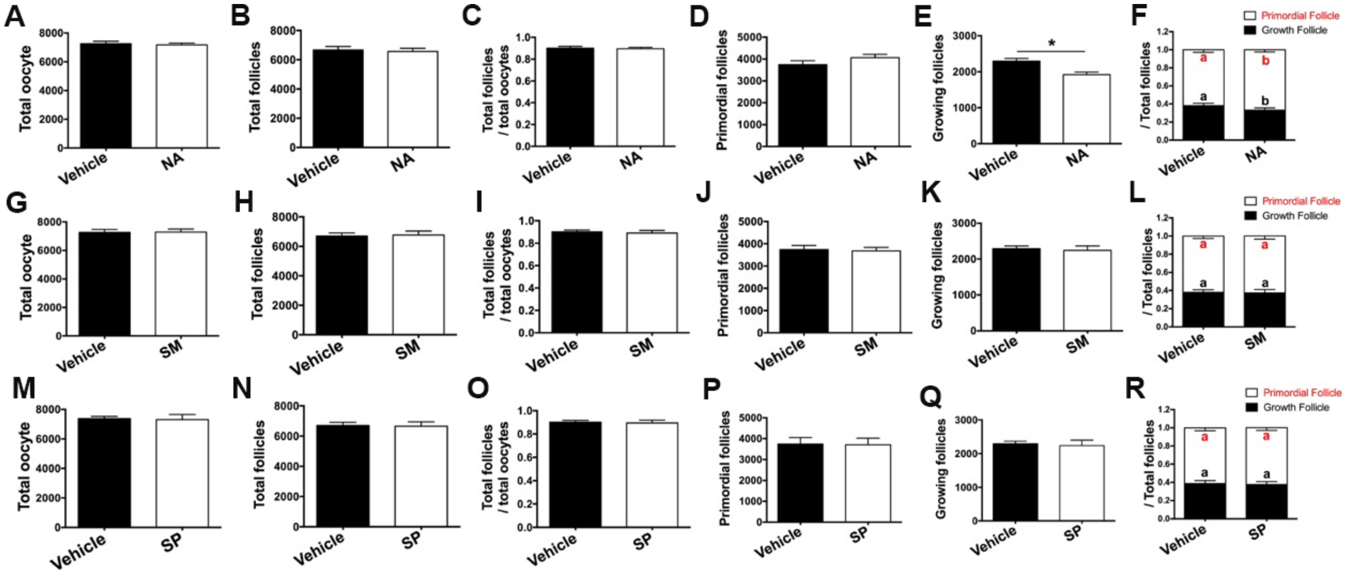

Supplementary Figure 2. The numbers of oocytes, follicles, PFs and GFs, and the proportions of PFs and GFs after inhibiting the three NOS isoforms. (**A**) The total number of oocytes, (**B**) the total number of follicles, (**C**) the total number of follicles/the total number of oocytes, (**D**) the number of PFs and (**E**) the number of GFs and (**F**) the numbers of PFs and GFs/the total number of follicles were counted in ovaries treated with the vehicle and eNOS inhibitor L-NAME (NA, 100 μM) for six days (n=6). (**G**) The total number of oocytes, (**H**) the total number of follicles, (**I**) the total number of follicles/the total number of oocytes, (**J**) the number of PFs and (**K**) the number of GFs and (**L**) the numbers of PFs and GFs/the total number of follicles were counted in ovaries treated with the vehicle and iNOS inhibitor SMT (SM, 1 mM) for six days (n=6). (**M**) The total number of oocytes, (**N**) the total number of follicles, (**O**) the total number of follicles/the total number of oocytes, (**P**) the number of PFs and (**Q**) the number of GFs and (**R**) the numbers of PFs and GFs/the total number of follicles were counted in ovaries treated with the vehicle and nNOS inhibitor spermidine (SP, 10 mM) for six days (n=6). * denotes statistical significance at $p < 0.05$. Different letters with the same color denote statistical significance at $p < 0.05$ (Red letters represent the proportions of PFs, while black letters represent the proportions of GFs).

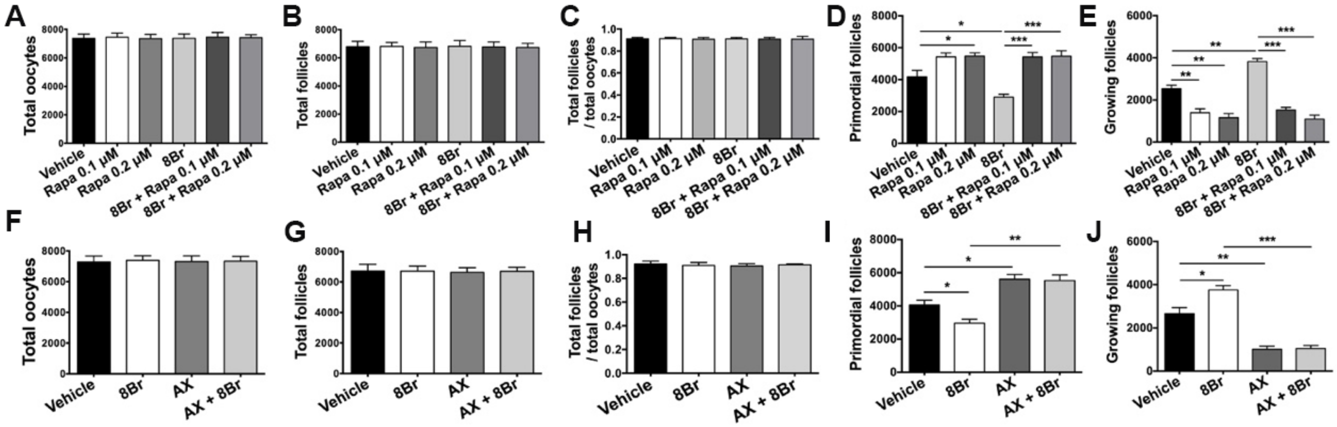

Supplementary Figure 3. The numbers of oocytes, follicles, PFs and GFs after simultaneously inhibiting mTOR/KIT and activating eNOS/cGMP/PKG. (**A**–**E**) Ovaries at 1 dpp were treated with the vehicle, 8Br (10 μM), the mTOR inhibitor rapamycin (Rapa) or 8Br + Rapa for six days (n=6). (**A**) The total number of oocytes, (**B**) the total number of follicles, (**C**) the total number of follicles/the total number of oocytes, (**D**) the number of PFs and (**E**) the number of GFs were counted. (**F**–**J**) Ovaries at 1 dpp were treated with vehicle, 8Br (10 μM), the KIT inhibitor Axitnib (AX, 5 μM) or 8Br + AX for six days (n=6). (**F**) The total number of oocytes, (**G**) the total number of follicles, (**H**) the total number of follicles/the total number of oocytes, (**I**) the number of PFs and (**J**) the number of GFs were counted. *, ** and *** denote statistical significance at $p < 0.05$, $p < 0.01$ and $p < 0.001$, respectively.

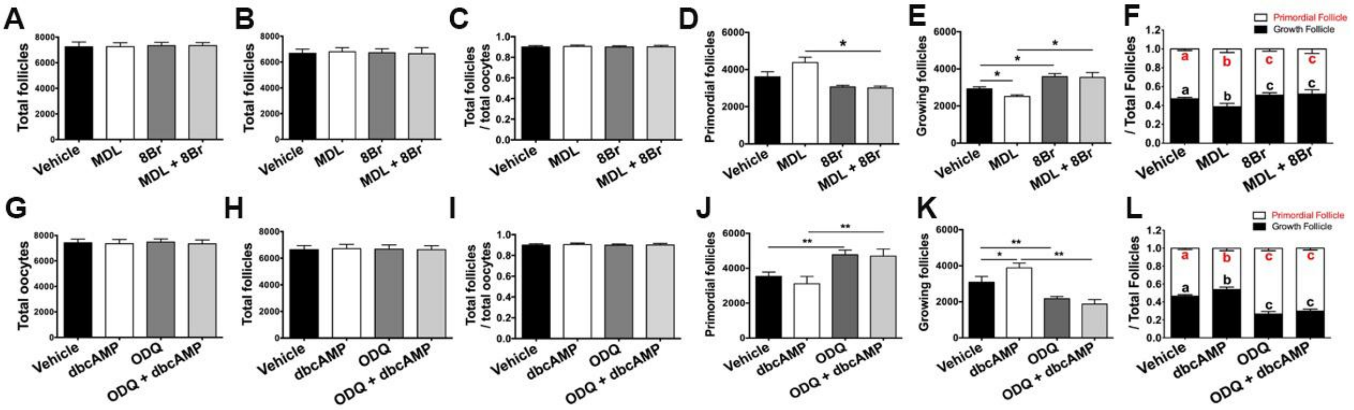

Supplementary Figure 4. The numbers of oocytes, follicles, PFs and GFs, and the proportions of PFs and GFs after altering cGMP and cAMP signals. (**A**–**F**) Ovaries at 1 dpp were treated with the vehicle, the adenylyl cyclase inhibitor MDL-12,330 (MDL, 5 μM), 8Br (10 μM), or MDL + 8Br for six days (n=6). (**A**) The total number of oocytes, (**B**) the total number of follicles, (**C**) the total number of follicles/the total number of oocytes, (**D**) the number of PFs, (**E**) the number of GFs and (**F**) the numbers of PFs and GFs/the total number of follicles were counted. (**G**–**L**) Ovaries at 1 dpp were treated with the vehicle, ODQ (1 μM), the cAMP analog dbcAMP (10 μM), or ODQ + dbcAMP for six days (n=6). (**G**) The total number of oocytes, (**H**) the total number of follicles, (**I**) the total number of follicles/the total number of oocytes, (**J**) the number of PFs, (**K**) the number of GFs and (**L**) the numbers of PFs and GFs/the total number of follicles were counted. * and ** denote statistical significance at $p < 0.01$ and $p < 0.001$, respectively. Different letters with the same color denote statistical significance at $p < 0.05$ (Red letters represent the proportions of PFs, while black letters represent the proportions of GFs).
