# Supplementary material for: The endothelial nitric oxide synthase/cyclic guanosine monophosphate/protein kinase G pathway activates primordial follicles
Source: Aging (Albany NY). 2020 Dec 3;13(1):1096–119. doi: 10.18632/aging.202235 (PMC7835019; doi:10.18632/aging.202235)
Supplement: Supplementary Tables [file aging-13-202235-s002.pdf]

SUPPLEMENTARY TABLES

Supplementary Table 1. Primers used in our study.

Genes	Forwards	Backwards
eNOS	5'-ATGCCCAACCCAAACCTTGT-3'	5'-AGAGGTGTCTGGGACTCACT-3'
GUCY1a1	5'TAAGAGGGGCAAAGTCTCTTCA-3'	5'-GCCCTTTTCCAAGCAGGACT-3'
GUCY1b1	5'-ATAAGAGGGGCAAAGTCTCTTCA-3'	5'-AGGGCAAACAGGGATGCTTA-3'
MVH	5'-CCTCCACCAGAGGATGAGGA-3'	5'-GGTGGTGCATCATGTCCAGA-3'
FOXL2	5'-AACACCGGAGAAACCAGACC-3'	5'-CGTAGAACGGGAACTTGGCT-3'
PKG	5'-CACCCTGCGGGATTTACAGT-3'	5'-CACCGAGCGATACTTGTCCA-3'
FBXW7	5'- TTCATTCCTGGAACCCAAAGA-3'	5'-TCCTCAGCCAAAATTCTCCAGTAC-3'
mTOR	5'-GTCGGCACTCCACTATCCTG-3'	5'-ATGGAGATCCTTGGCACACC-3'
β-actin	5'-AGAGGGAAATCGTGCGTGAC-3'	5'-CAATAGTGATGACCTGGCCGT-3'

Supplementary Table 2. Primary antibodies used in our study.

Antibody	Source	Cat.	Dilution	
		number	IF/IHC	WB
eNOS	Novus	NB300-500	1:100	
nNOS	CST	4231	1:100	
iNOS	CST	13120	1:100	
PKG	CST	3248	1:50	
mTOR	Gentex	GT 649	1:50	
mTOR	CST	2983		1:1000
MSY2	Santa Cruz	Sc-21316	1:100	
FOXO3a	CST	12829	1:50	
FBXW7	Thermo Scientific	40-1500	1:50	1:1000
Flag	Thermo Scientific	F2555	1:100	
GAPDH	CST	2118		1:1000

IF, immunofluorescence; IHC, immunochemistry staining; WB, Western blotting.
